# Urban sensing in the era of large language models

**DOI:** 10.1016/j.xinn.2024.100749

**Published:** 2025-01-06

**Authors:** Ce Hou, Fan Zhang, Yong Li, Haifeng Li, Gengchen Mai, Yuhao Kang, Ling Yao, Wenhao Yu, Yao Yao, Song Gao, Min Chen, Yu Liu

**Affiliations:** 1 Institute of Remote Sensing and Geographical Information System, School of Earth and Space Sciences, Peking University, Beijing 100871, China; 2 Department of Electronic Engineering, Tsinghua University, Beijing 100084, China; 3 School of Geosciences and Info-Physics, Central South University, Changsha, Hunan 410083, China; 4 Xiangjiang Laboratory, Changsha, Hunan 410205, China; 5 Department of Geography and the Environment, The University of Texas at Austin, Austin, TX 78712, USA; 6 State Key Laboratory of Resources and Environmental Information System, Institute of Geographic Sciences and Natural Resources Research, Chinese Academy of Sciences, Beijing 100101, China; 7 School of Geography and Information Engineering, China University of Geosciences, Wuhan 430074, China; 8 Center for Spatial Information Science, The University of Tokyo, Chiba 113-8654, Japan; 9 Department of Geography, University of Wisconsin-Madison, Madison, WI 53706, USA; 10 Key Laboratory of Virtual Geographic Environment (Ministry of Education of PRC), Nanjing Normal University, Nanjing 210023, China; 11 Department of Civil and Environmental Engineering, The Hong Kong University of Science and Technology, Hong Kong SAR, China

## Abstract

Urban sensing has become increasingly important as cities evolve into the centers of human activities. Large language models (LLMs) offer new opportunities for urban sensing based on commonsense and worldview that emerged through their language-centric framework. This paper illustrates the transformative impact of LLMs, particularly in the potential of advancing next-generation urban sensing for exploring urban mechanisms. The discussion navigates through several key aspects, including enhancing knowledge transfer between humans and LLM, urban mechanisms awareness, and achieve automated decision-making with LLM agents. We emphasize the potential of LLMs to revolutionize urban sensing, offering a more comprehensive, efficient, and in-depth understanding of urban dynamics, and also acknowledge challenges in multi-modal data utilization, spatial-temporal cognition, cultural adaptability, and privacy preservation. The future of urban sensing with LLMs lies in leveraging their emerged intelligent and addressing these challenges to achieve more intelligent, responsible, and sustainable urban development.

## Introduction

**Figure 1:**
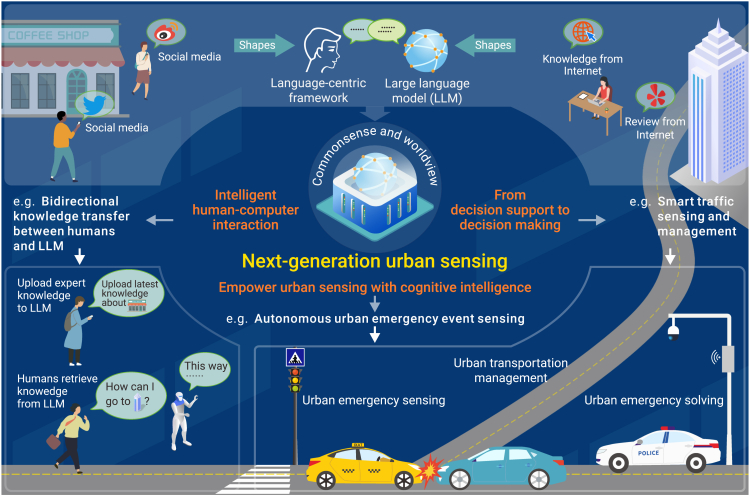
LLMs have been driving the evolution of next-generation urban sensing

Urban sensing integrates and interprets multi-modal data on urban environments and human activities.^1^^,^^2^ Current urban sensing technologies focus on diverse urban phenomena, such as monitoring traffic flow dynamics, assessing air quality, or tracking the trajectories of human activities. However, they fall short of collaboratively sensing the interrelationships among these urban phenomena, such as the interaction between humans and the built environment.^3^ This limitation significantly hinders urban sensing’s ability to understand the urban mechanisms, thereby constraining urban sensing’s further development.^4^^,^^5^ Large language models (LLMs), represented by GPT, Gemini, and LLaMA, offer promising breakthroughs for addressing this challenge through their emerging commonsense and worldview, which encompass an inherent understanding of the world including time, space, physics, social interactions, and emotions, as well as the capacity to reason within these contexts.^6^ These commonsense and worldview provide LLMs with unique latent guidance in understanding the urban mechanisms through various urban phenomena, thus providing the prerequisites for forming next-generation urban sensing.^7^^,^^8^ This paper explores how LLMs can significantly advance the development of next-generation urban sensing (as illustrated in Figure 1) and the potential challenges during this transformative era.

## Commonsense and worldview shape LLMs as the transformative role toward next-generation urban sensing

The language-centric framework is fundamental for LLMs distilling commonsense and worldview from big data. Among various modalities, language excels in its efficiency and comprehensiveness for encoding knowledge, serving as the foundation for the emergence of commonsense and worldview in LLMs. Practically, the high availability of language data on the Internet provides rich abundant resources for training LLMs. Language also encapsulates complex concepts, facts, and opinions more effectively than other modalities like visual or auditory. Theoretically, the “global workspace theory” identifies language as a pivotal medium for aligning multi-modal spatiotemporal data.^9^ Language facilitates multi-modal data to be transformed into a unified feature space. This crucial role highlights its importance in integrating and summarizing diverse data modalities.^10^

The emergence of commonsense and worldview in LLMs revolutionizes urban sensing paradigms toward a new generation characterized by interactivity, cognitive intelligent, and automated decision-making.

Firstly, next-generation urban sensing transforms human-computer interaction by enabling bidirectional knowledge transfer. Urban sensing relies not only on commonsense and worldview but also on domain-specific knowledge to enhance its ability to deal with domain issues. Technologies such as retrieval-augmented generation enable LLMs to incorporate expert urban science knowledge, thereby enhancing the accuracy and professionalism of urban sensing. In addition, integrating commonsense and worldview improves LLMs’ communicative abilities, enabling them to articulate urban dynamics with smooth and human-like interaction. This ability improves the efficiency of humans in acquiring knowledge from LLMs.

Secondly, commonsense and worldview empower urban sensing with cognitive intelligence, allowing next-generation urban sensing to aware urban mechanisms. Current urban sensing methods often operate within rigid and predefined constraints, such as managing traffic based on pre-configured holiday and weekend data. This limitation hinders the model’s adaptability for unforeseen circumstances. Integrating commonsense and worldview knowledge provides models with a comprehensive contextual understanding of urban environments, such as general traffic flow patterns, the effects of weather on travel, and the influence of social events on human mobility. Such knowledge serves as implicit constraints, enabling models to response complex emergency events based on fundamental urban mechanisms. In practical applications, this capability allows next-generation urban sensing to accurately and flexibly detect urban anomalies. For example, LLM-enhanced urban sensing improves the efficiency and accuracy of emergency management by identifying high-risk areas for incidents like traffic accidents or public gatherings, enhancing responsiveness in urban emergencies events through the urban mechanisms they learned.

Finally, embedding commonsense and worldview into agents shifts urban sensing from decision support to autonomous decision-making. Commonsense and worldview provide LLMs with cognitive and judgment capabilities, enabling them to understand and respond to their urban sensing outcomes. This ability ensures that LLMs can be closely integrated with the agents on the executing side, collectively forming the decision-making and feedback loop system that achieves automated cycles from urban sensing to strategic response. For example, an LLM-based traffic sensing and management system can autonomously detect congestion and command agents to manage traffic flow through dynamic regulation strategies. Based on current traffic flow patterns and signal control data, LLMs predict high-risk areas for future congestion and formulate appropriate traffic mitigation strategies. Agents then implement these strategies, which may include adjusting signal phase timings or initiating emergency traffic management measures to alleviate potential congestion proactively.

Future research should concentrate on the in-depth exploration and application of commonsense and worldview within LLMs. Theoretically, future studies should reveal the underlying mechanisms driving the emergence of commonsense and worldview in LLMs, such as how training data diversity and algorithmic innovations influence these abilities. Efforts should also work on enhancing LLMs’ capacity to exhibit more comprehensive and profound commonsense and worldview, pushing the boundaries of artificial intelligence. Practically, researchers should actively explore the broad application scenarios for LLMs’ commonsense and worldview in urban sensing. For example, LLM-driven intelligent platforms could be developed to provide tailored solutions across domains such as urban planning, traffic management, and environmental protection. Moreover, LLM-driven virtual town simulations are also promising for assessing the impacts of policy interventions and external factors like climate change, thereby supporting more effective and sustainable strategies.

## Challenges for LLMs in urban sensing

The commonsense and worldview exhibited by LLMs offer unprecedented transformative potential for next-generation urban sensing. However, these capabilities heavily rely on the large scale language data in LLM’s training processes. The “inductive biases” inherent in these inputted data inevitably shape the commonsense and worldview, leading to mismatch with the ideals of human society. Consequently, leveraging LLMs to develop next-generation urban sensing systems still confronts a series of challenges.

The first challenge lies in effectively integrating diverse multi-modal data in urban sensing. LLMs trained on language data often encounter incompatibility with non-textual modalities like trajectory and graph data, limiting their ability to assimilate broader and more multi-dimensional urban information. Although multi-modal alignment technologies offer promising solutions, aligning diverse data types remains a highly challenging task due to differences in their organization structure and diverse information.

The second challenge is the absence of robust spatiotemporal cognition, which is the understanding of space, time, dimensions, and relationships within a geographical context. Due to the insufficient emphasis on data’s spatiotemporal characteristics during training, LLMs cannot inherently process and understand spatiotemporal data. Although LLMs can process textual descriptions of spatiotemporal relationships, their understanding relies on language rather than real perception, like humans or spatially focused systems. LLMs’ low sensitivity to numeric values further hinders accurate spatiotemporal perception and prediction. These problems limit their effectiveness in accurate measurements or quantitative reasoning and poses challenges for urban sensing tasks, such as capturing changes across different locations and time periods. For example, LLMs frequently fail in precise spatial cognition, such as accurately identifying cities within a 100-km radius north of Beijing. Similarly, they also face challenges with long-term time-series data predictions, generating hallucinations that appear plausible in format but deviate significantly from logical or commonsense expectations.

Cultural and value diversity among different groups poses a third challenge for LLMs in capturing and interpreting various perspectives on the same urban phenomena. Recognizing individual differences within LLMs from a human perspective is crucial, as human attitudes toward issues are shaped by religious, cultural, and ideological factors, leading to varied interpretations across various contexts. For instance, urban graffiti can be interpreted either as a disruption to community order or as a vital expression of street culture and ideological liberation, depending on cultural and social context. When mainstream perspectives dominate, minority voices will be marginalized, resulting in potential discrimination and systemic biases in LLMs.

Constructing an effective privacy-preserving framework is the last challenge for LLMs in urban sensing. Since LLM training relies on real human activity data, such as social media records, the models’ complex architectures make it difficult to fully control the generated outputs. Carefully crafted adversarial prompts can exploit model vulnerabilities, leading to potential leakage of original training data and raising significant privacy concerns. This challenge undermines the broader adoption of LLMs in urban sensing and erodes public trust in their applications.

Addressing these challenges will enhance the diversity and robustness of training data and refine the model training process to seek effective solutions. Introducing continuous learning mechanisms and reinforcement learning with human feedback becomes crucial and urgent for facing these challenges. By continuously updating LLMs with the latest language data and incorporating human guidance, the LLM’s dynamic adaptability, social sensitivity, and privacy protection capabilities are significantly enhanced. This approach also allows LLMs to capture and respond to society’s evolving needs while effectively addressing the dynamics of urban environments more accurately. These efforts are essential for advancing urban sensing technologies toward greater fairness and inclusivity, ensuring that LLM-driven urban sensing benefits all segments of society and supports sustainable urban development.

## Conclusion

This article examines the potential applications and key challenges of LLMs in urban sensing, particularly in shaping next-generation urban sensing. As cities become central to human activity, the need for effective and responsive urban sensing grows. LLMs, with their commonsense worldview, offer a significant improvement over current methods in understanding and interpreting urban environments.

Looking ahead, we are in this transformative era, where it is crucial to develop a collaborative ecosystem where technological advancements and human-centric considerations converge. This convergence will enhance the technical robustness of urban sensing while grounding it in diverse human experiences and social values. By unlocking the full potential of LLMs, we can pave the way for a new era of urban sensing that is not only more efficient and intelligent but also more adaptable and inclusive, contributing to the creation of smarter, more sustainable urban environments.

## Acknowledgments

This work was supported by the 10.13039/501100001809National Natural Science Foundation of China under grants 42430106 and 42371468, and the Public Policy Research Funding Scheme of the Chief Executive’s Policy Unit of Hong Kong under project 2023.A7.025.23A. This work was supported by the High-performance Computing Platform of Peking University (hpc2306190166).

## Declaration of interests

The authors declare no competing interests.
